# Invasive Fungal Sinusitis in Patients With Hematological Malignancies: A 20-Year Study From a Tertiary Academic US Hospital System

**DOI:** 10.1093/ofid/ofag304

**Published:** 2026-05-15

**Authors:** Tejas S Athni, Carolyn B Strauch, Victor Kovac, Esther Arbona-Haddad, Inia Perez Villa, Simran Gupta, Muneerah M Aleissa, Alexis D Liakos, Alexandra Tong, Rahul S Vedula, Alice Z Maxfield, Regan W Bergmark, Amy C Sherman

**Affiliations:** Harvard Medical School, Boston, Massachusetts, USA; Department of Epidemiology, Harvard T.H. Chan School of Public Health, Boston, Massachusetts, USA; Department of Medicine, Beth Israel Deaconess Medical Center, Boston, Massachusetts, USA; Division of Infectious Diseases, Brigham and Women's Hospital, Boston, Massachusetts, USA; Division of Infectious Diseases, Brigham and Women's Hospital, Boston, Massachusetts, USA; Division of Infectious Diseases, Brigham and Women's Hospital, Boston, Massachusetts, USA; Harvard Medical School, Boston, Massachusetts, USA; Division of Infectious Diseases, Brigham and Women's Hospital, Boston, Massachusetts, USA; Division of Infectious Diseases, Brigham and Women's Hospital, Boston, Massachusetts, USA; College of Pharmacy, Department of Pharmacy Practice, Princess Nourah Bint Abdulrahman University, Riyadh, Saudi Arabia; Division of Infectious Diseases, Brigham and Women's Hospital, Boston, Massachusetts, USA; Division of Infectious Diseases, Brigham and Women's Hospital, Boston, Massachusetts, USA; Department of Medical Oncology, Dana Farber Cancer Institute, Boston, Massachusetts, USA; Harvard Medical School, Boston, Massachusetts, USA; Division of Otolaryngology-Head and Neck Surgery, Brigham and Women's Hospital, Boston, Massachusetts, USA; Harvard Medical School, Boston, Massachusetts, USA; Division of Otolaryngology-Head and Neck Surgery, Brigham and Women's Hospital, Boston, Massachusetts, USA; Harvard Medical School, Boston, Massachusetts, USA; Division of Infectious Diseases, Brigham and Women's Hospital, Boston, Massachusetts, USA; Department of Medical Oncology, Dana Farber Cancer Institute, Boston, Massachusetts, USA

**Keywords:** hematological malignancy, invasive fungal sinusitis, rhino-orbital-cerebral mucormycosis

## Abstract

**Background:**

Invasive fungal sinusitis (IFS) profoundly impacts individuals with hematological malignancies, with rapid progression and high mortality rates. In an evolving therapeutic landscape, outcomes for patients with hematological malignancies who develop IFS warrant further evaluation.

**Methods:**

We performed a descriptive case series and retrospective review of patients with hematologic malignancies who developed proven IFS (per European Organization for Research and Treatment of Cancer/Mycoses Study Group criteria) between 2005 and 2024 at a tertiary academic center. Clinical data and outcomes were collected from electronic medical records. Statistical analyses included Fisher exact tests, *t* tests, or Wilcoxon rank-sum tests. Kaplan-Meier curves were used to estimate survival probability, and log-rank tests were compared across groups.

**Results:**

Thirty-one patients with confirmed IFS were identified. Most patients had acute myeloid leukemia (61.3%) and received cytotoxic chemotherapy (96.8%). Targeted oncologic therapies included BCL2 inhibitors (29.0%), FLT3 inhibitors (19.4%), antibody-based therapies (12.9%), BTK inhibitors (6.4%), and antibody–drug conjugates (6.4%). The most common fungal isolates were *Aspergillus* (22.6%), *Fusarium* (16.1%), and *Rhizopus* (9.7%). Amphotericin B (96.8%) and voriconazole (71.0%) were frequently used, and newer antifungals included isavuconazole (38.7%), olorofim (3.3%), and fosmanogepix (3.3%). Necrotic tissue (45.2%) and mucosal thickening (87.1%) were common on sinonasal endoscopy and computed tomography scan, respectively. Orbital (25.8%) and intracranial (9.7%) extension occurred in a minority. Most patients underwent functional endoscopic sinus surgery (80.6%). Serum fungal biomarkers were infrequently positive, with (1,3)-ß-D-glucan in 19.4% and galactomannan in 9.7%. Overall mortality was 71.0% at the end of the study period, with a median survival (interquartile range) of 101 (21–299) days. Mortality was 22.6% at 1 month, 25.8% at 2 months, 32.3% at 3 months, 41.9% at 6 months, 54.8% at 1 year, 58.1% at 2 years, and 64.5% at 3 years.

**Conclusions:**

Despite novel therapies and aggressive surgical interventions, patients with IFS had high mortality.

Invasive fungal sinusitis (IFS) is a potentially fatal opportunistic infection that largely impacts immunocompromised individuals, characterized by rapid disease progression, slow response to medical therapy, and high mortality rates [1, 2]. Patients who are immunocompromised due to hematological malignancies are especially susceptible, given host immune defects due to the malignancy itself as well as the immunosuppressive effects of oncology treatments. Prognosis of IFS is poor, with 3-month mortality estimated at 48% [3] and 1-year mortality estimated at 68% in patients with hematologic malignancies [4].

The sinonasal tract, as the first point of contact for inhaled air, is often exposed to bacteria, fungi, viruses, and environmental insults. For immunocompromised patients, these insults can lead to rhinologic disease that can be fatal [5–7]. IFS cases require multidisciplinary care from rhinologists/endoscopic skull base surgeons, infectious disease specialists, immunologists, and oncologists. As IFS remains relatively rare, creating large-scale clinical trials to investigate novel therapeutics is difficult, with many regulatory barriers [8].

The clinical presentation of IFS often has nonspecific and deceptively subtle symptoms such as nasal obstruction, rhinorrhea, and fever, which often delays the recognition of the disease. Only in later stages do more obvious symptoms present, such as black eschar on the nasal mucosa or palate, vision changes, ophthalmoplegia, cranial neuropathies, and facial numbness. Histologically, fungal elements can breach mucosal barriers and invade blood vessels, leading to tissue necrosis [9, 10]. IFS tends to be caused by a dominant fungal pathogen but may involve a combination of fungal species [11].

Patients with hematological malignancies are particularly vulnerable to IFS owing to prolonged neutropenia, intrinsic and iatrogenic immunosuppression, and mucosal injury. Novel anticancer therapies, such as BCL2 inhibitors (venetoclax), tyrosine kinase inhibitors targeting FLT3 (gilteritinib, midostaurin) and BTK (zanubrutinib), antibody-based therapies targeting CD20 (obinutuzumab), and antibody–drug conjugates targeting CD22 (inotuzumab ozogamicin) or CD30 (brentuximab vedotin), have improved oncologic survival rates. However, these drugs result in immune dysregulation, such as inhibited neutrophil oxidative burst formation, regulatory T-cell suppression, T-cell exhaustion and altered checkpoint expression, dampening of dendritic cell immunosurveillance and pro-inflammatory cytokine secretion, and capillary leak syndrome [12–17], in addition to profound neutropenia and lymphopenia. All of these mechanisms may predispose patients to developing IFS or exacerbate progression of IFS.

Overall mortality remains high despite use of systemic antifungal therapy for IFS [18]. However, novel antifungal agents have been developed recently with additional antifungal therapies in the development pipeline [19]. Newer-generation therapies such as dihydroorotate dehydrogenase inhibitors (olorofim), Gwt1 protein inhibitors (fosmanogepix), and triazoles (isavuconazole) offer broader-spectrum coverage, more favorable toxicity profiles, improved pharmacokinetic profiles (longer half-life, enabling once-daily dosing), and greater in vitro activity against resistant fungal strains as compared with older generations of therapies (eg, amphotericin B, voriconazole, terbinafine, micafungin, flucytosine) [20]. The impact of this evolving cascade of anticancer and antifungal therapies on IFS has yet to be characterized.

Other studies have described the characteristics of IFS in hematologic malignancy populations, but hematologic malignancy comprised only a small part of their overall study population [21–25]. Our study therefore aims to characterize the epidemiology, medical and surgical factors, and landscape of IFS in patients with hematological malignancies.

## METHODS

We conducted a descriptive case series and retrospective review of proven IFS in adult patients with hematologic malignancies between 2005 and 2024 at a large, tertiary academic center. Patients were identified from the Mass General Brigham's Research Patient Data Registry (RPDR) and via clinician identification from the hematopoietic cell transplant (HCT), oncology, and otolaryngology inpatient or outpatient services at Brigham and Women's Hospital and Dana-Farber Cancer Institute. Definitions of IFS were based on 2020 European Organization for Research and Treatment of Cancer and the Mycoses Study Group (EORTC/MSG) consensus criteria [26]. Briefly, proven IFS in the EORTC/MSG criteria is defined by histopathologic or cytopathologic evidence of fungal elements with tissue damage, recovery of mold from a normally sterile site, blood culture positive for mold in a compatible clinical context, or detection of fungal DNA by polymerase chain reaction (PCR) in tissue specimens [26]. Patients were included if they were adults who were 18 years and older at the time of IFS diagnosis and had proven IFS, a confirmed hematologic malignancy, and available electronic medical record (EMR) data. Patients were followed until death or resolution of fungal disease. This study was approved by the Mass General Brigham Institutional Review Board (IRB #2023P000909).

Patient demographics (age, gender, body mass index, race); laboratory results at the time of IFS diagnosis (white blood cell count, absolute neutrophil count, absolute lymphocyte count, platelets, hematocrit, immunoglobulin [Ig] G); oncologic history (type of hematologic malignancy, treatments received); mycologic (histopathology, fungal markers, cultures, molecular diagnostics, antifungals received), radiographic (computed tomography [CT] and magnetic resonance imaging closest to date of diagnosis, laterality, imaging features such as orbital extension, intracranial extension, bony erosion, and fat stranding), and surgical (laterality, extent) data; sinonasal endoscopy and otorhinolaryngology physical exam findings (cranial nerve involvement, mucosal paleness, edema, necrosis, ulcer, crusting, purulence); and mortality outcomes (death status, cause, date) were collected from the EMR. For laboratory results, the following definitions were used: neutropenia <1500 cells/µL, severe neutropenia <500/µL, lymphopenia <1000 cells/µL, severe lymphopenia <500 cells/µL, thrombocytopenia <150 000 cells/µL, severe thrombocytopenia <50 000 cells/µL, anemia <36% hematocrit, and hypogammaglobulinemia IgG <600 mg/dL. Lack of documentation of any of the above findings was interpreted as an absence of these findings, unless otherwise indicated. Lund-McKay scores [27] were computed from CT face/sinus imaging by otolaryngology specialists and checked by a second reviewer.

Frequencies were reported for categorical variables. Percentages for table subcategories are calculated with a denominator of the broader n = 31 cohort rather than as a subset of the specific category under which they fall. Medians with interquartile ranges were reported for continuous variables. The Fisher exact test was used to compare categorical variables where at least 1 cell had a value <5. *T* tests or Wilcoxon rank-sum tests (Mann-Whitney *U* tests) were used to compare continuous variables. Mortality was descriptively calculated at the following time points: 1 month, 2 months, 3 months, 6 months, 1 year, 2 years, and 3 years. Cumulative mortality was plotted. A Kaplan-Meier curve was plotted to visually depict survival probability, with 95% CIs generated using Greenwood's formula and a log-log transformation, and was truncated at 24 months. R, version 4.5.0, was used for all computation.

A set of exploratory subgroup analyses assessing 6-month mortality were performed. Data were stratified across newer vs older antifungal groups, *Aspergillus* vs non-*Aspergillus* fungal isolate, early (≤1 day) vs delayed surgical intervention (>1 day), receipt of hematopoietic stem cell transplant, and receipt of steroids. Newer antifungals were defined as medications approved by the Food and Drug Administration (FDA) in 2015 (isavuconazole) or still in unapproved, investigational status (olorofim and fosmanogepix). Older antifungals consisted of medications approved by the FDA before 2015 (amphotericin B, voriconazole, posaconazole, terbinafine, micafungin, caspofungin, and flucytosine). Six-month mortality percentages were compared descriptively using the chi-square test or Fisher exact test.

## RESULTS

Thirty-one patients with confirmed IFS diagnosed between 2005 and 2024 were included, with characteristics described in Table 1. The median age was 59 years, and 83.9% of patients were White. Most patients had an underlying diagnosis of acute myeloid leukemia (AML; 70.0%) and were diagnosed in the latter 2015–2024 decade (83.9%) (Supplementary Figure 1). Of patients with available lab data, most had marked neutropenia (82.8%), lymphopenia (79.3%), thrombocytopenia (96.6%), and anemia (93.1%), while a smaller fraction of patients had hypogammaglobulinemia (40.9%) at the time of IFS diagnosis. Within the 6 months before IFS, nearly all patients in the study cohort received conventional cytotoxic chemotherapy (96.8%), including antimetabolites (64.5%), anthracyclines (45.2%), hypomethylating agents (19.4%), and alkylating agents (16.1%). Immunosuppressant therapy was initiated in 38.7% of patients. Targeted oncologic therapies included BCL2 inhibitors such as venetoclax (29.0%); FLT3 inhibitors such as gilteritinib (9.7%), midostaurin (6.5%), and sorafenib (3.2%); BTK inhibitors such as ibrutinib (3.2%) and zanubrutinib (3.2%); antibody-based therapies such as rituximab (6.5%), obinutuzumab (3.2%), and alemtuzumab (3.2%); and antibody–drug conjugates such as brentuximab vedotin (3.2%) and inotuzumab ozogamicin (3.2%).

**Table 1.** Demographics, Baseline Labs, and Oncologic History of Patients With Hematologic Malignancies and Invasive Fungal Sinusitis

Characteristic^a^	Value
Overall, No.	31
Demographics	
Age, median (IQR), y	59 (51–67)
Male, No. (%)	16 (51.6)
White, No. (%)	26 (83.9)
Black, No. (%)	2 (6.5)
BMI, median (IQR), kg/m^2^	23.6 (20.6–28.6)
Labs at time of IFS diagnosis, median (IQR)	
White blood cell count, 10^3^ cells/µL	0.56 (0.21–3.70)
Absolute neutrophil count, 10^3^ cells/µL	0.01 (0.00–0.50)
Absolute lymphocyte count, 10^3^ cells/µL	0.42 (0.07–0.76)
IgG, mg/dL	645 (525–887)
Hematocrit, %	22.7 (20.8–27.4)
Platelets, 10^3^ cells/µL	29.0 (11.0–52.0)
Hematological malignancy diagnosis, No. (%)^b^	
Acute myeloid leukemia	19 (61.3)
Chronic lymphocytic leukemia	2 (6.5)
Follicular lymphoma^c^	2 (6.5)
Myelodysplastic syndrome^d^	2 (6.5)
Large cell lymphoma	1 (3.2)
Acute lymphoblastic leukemia	1 (3.2)
Prolymphocytic leukemia^e^	1 (3.2)
Hodgkin's lymphoma	1 (3.2)
Myelofibrosis	1 (3.2)
Polycythemia vera	1 (3.2)
Oncologic treatment, No. (%)^f,g^	
Cytotoxic chemotherapy	30 (96.8)
Antimetabolites	20 (64.5)
Cytarabine	15 (48.4)
Fludarabine	7 (22.6)
Methotrexate	4 (12.9)
Anthracyclines	14 (45.2)
Daunorubicin	9 (29.0)
Mitoxantrone	3 (9.7)
Doxorubicin	2 (6.5)
Hypomethylating agents	6 (19.4)
Azacitidine	5 (16.1)
Decitabine	1 (3.2)
Alkylating agents	5 (16.1)
Cyclophosphamide	2 (6.5)
Melphalan	1 (3.2)
Bendamustine	1 (3.2)
Busulfan	1 (3.2)
Hematopoietic cell transplantation	16 (51.6)
Allogeneic	14 (45.2)
Autologous	2 (6.5)
Targeted therapies and tyrosine kinase inhibitors	13 (41.9)
Venetoclax (BCL2-i)	9 (29.0)
Gilteritinib (FLT3-i)	3 (9.7)
Midostaurin (FLT3-i)	2 (6.5)
Sorafenib (FLT3-i)	1 (3.2)
Ibrutinib (BTK-i)	1 (3.2)
Zanubrutinib (BTK-i)	1 (3.2)
Immunosuppressants	12 (38.7)
Steroids	7 (22.6)
Tacrolimus	7 (22.6)
Sirolimus	3 (9.7)
Mycophenolate mofetil	1 (3.2)
Antibody-based therapies	4 (12.9)
Rituximab (anti-CD20)	2 (6.5)
Anti-thymocyte globulin	2 (6.5)
Obinutuzumab (anti-CD20)	1 (3.2)
Alemtuzumab (anti-CD52)	1 (3.2)
Antibody–drug conjugates	1 (3.2)
Brentuximab vedotin (anti-CD30)	1 (3.2)
Inotuzumab ozogamicin (anti-CD22)	1 (3.2)

Abbreviations: BCL2-i, B-cell lymphoma-2 inhibitor; BMI, body mass index; BTK-i, Bruton tyrosine kinase inhibitors; IFS, invasive fungal disease; IgG, immunoglobulin G; IQR, interquartile range.

^a^All cases included were “proven invasive fungal disease” according to European Organization for Research and Treatment of Cancer and the Mycoses Study Group Education and Research Consortium (EORTC/MSGERC) definitions [26].

^b^Hierarchical diagnosis assignment rules were used such that if a patient had AML arising from MDS, they were counted as AML only.

^c^One patient with follicular lymphoma also developed treatment-related AML (secondary to follicular lymphoma).

^d^One patient had aplastic anemia that transformed to MDS.

^e^One patient had prolymphocytic leukemia due to treatment-related CLL.

^f^Percentages for subcategories are reported as a percentage of the overall total (n = 31 patients) and not of the total in the parent “oncologic treatment” category in which they nested.

^g^Patients can be on multiple therapies.

Half of patients received a hematopoietic cell transplant (HCT; 51.6%), most of which were allogeneic (45.2%); few were autologous (6.5%). The median time from HCT to IFS diagnosis (interquartile range [IQR]) was 341 (189–796) days. Of note, in those individuals who had undergone HCT, 4 patients had GVHD, 3 of whom were receiving steroids for at least 3 weeks within the past 60 days from the diagnosis of IFS, 1 patient had graft failure after HCT at the time of IFS diagnosis, and 8 patients had relapsed disease, 6 of whom were receiving salvage chemotherapy at the time of IFS diagnosis.

**Figure 1.**
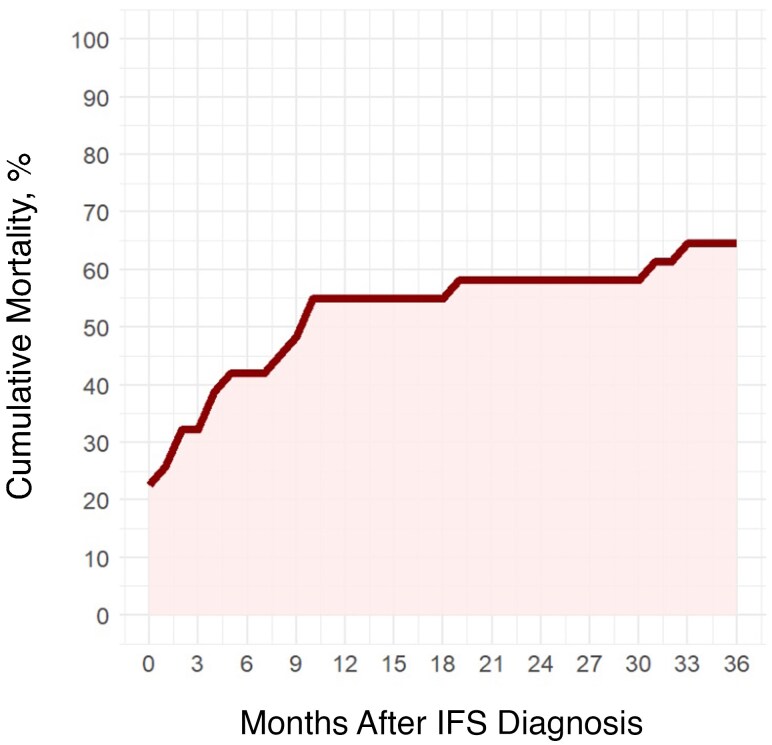
Cumulative mortality of IFS patients by month after fungal diagnosis. Abbreviation: IFS, invasive fungal sinusitis.

Overall mortality in the cohort was 71.0% at the conclusion of the study period. Mortality was 22.6% at 1 month, 25.8% at 2 months, 32.3% at 3 months, 41.9% at 6 months, 54.8% at 1 year, 58.1% at 2 years, and 64.5% at 3 years. Cumulative mortality over time can be seen in Figure 1. The estimated Kaplan-Meier curve can be seen in Supplementary Figure 2. The median time from IFS diagnosis to death (IQR) was 101 (21–299) days (Table 2). A quarter of the cohort (25.8%) had orbital extension, and of these individuals, 62.5% died before 3 months. Only 3 patients had fungal intracranial extension (9.7%), all of whom had concurrent orbital extension.

**Table 2.** Mortality Outcomes

Characteristic	Value
Overall, No.	31
Follow-up time from IFS diagnosis, median (IQR), d	209 (31–398)
Overall mortality at end of follow-up, No. (%)	
Death	22 (71.0)
Survival	8 (25.8)
Unknown	1 (3.2)
Cause of death, No. (%)^a^	
Fungal disease	9 (29.0)
Progressive malignancy	8 (25.8)
Other nonfungal infection	7 (22.6)
Other	6 (19.4)
Disease relapse of malignancy	6 (19.4)
Graft-vs-host disease	1 (3.2)
Time from IFS diagnosis to death by cause, median (IQR), d	
Fungal disease	22 (18–34)
Progressive malignancy	134 (31–260)
Other nonfungal infection	155 (25–304)
Other	295 (155–580)
Disease relapse of malignancy	202 (96–296)
GVHD	580
Overall mortality by timing of surgical intervention, No. (%)	
≤1 d of IFS diagnosis (n = 15)	7 (46.7)
>1 d of IFS diagnosis (n = 8)	7 (87.5)
Time from IFS diagnosis to death, median (IQR), d	101 (21–299)

Abbreviations: IFS, invasive fungal disease; IQR, interquartile range.

^a^Patients could have >1 cause of death.

Clinically, 54.8% of patients had IFS localized to the sinuses only, and 45.2% had disseminated fungal infections (Table 3). A range of diagnostic tests were used to identify the causative fungus, most commonly biopsy (93.5%) and histopathology (90.3%), but also including microbiological data (64.5%), molecular data (19.4%), and autopsy (6.5%). The most common fungal isolates were *Aspergillus* (22.6%), *Fusarium* (16.1%), and *Rhizopus* (9.7%) species, with rarer cases including *Curvularia* (6.7%), *Mucor* (6.7%), *Scedosporium* (3.2%), *Penicillium* (3.2%), *Lichtheimia* (3.2%), *Cunninghamella* (3.2%), *Alternaria* (3.2%), and *Rigidoporus* (3.2%). Most patients had not received antifungal prophylaxis before IFS (93.6%). Liposomal amphotericin B (96.8%) and voriconazole (71.0%) were the most common initial antifungal treatments. Novel antifungal agents used as additional therapy included olorofim (3.3%) and fosmanogepix (3.3%).

**Table 3.** Fungal Data, Diagnostics, and Antifungal Treatment

Characteristic	Frequency, No. (%)
Overall, No.	31
Extent of infection	
Localized	17 (54.8)
Disseminated	14 (45.2)
Diagnostic technique used	
Biopsy	29 (93.5)
Histopathology	28 (90.3)
Autopsy	2 (6.5)
Microbiological data	20 (64.5)
Molecular data	6 (19.4)
Serum (1,3)-ß-D-glucan	
Positive (≥80 pg/mL)	6 (19.4)
Negative (<60 pg/mL)	20 (64.5)
Indeterminate (60–79 pg/mL)	2 (6.5)
Galactomannan index^a^	
Positive (>0.5)	3 (9.7)
Negative (<0.5)	25 (80.6)
Fungus cultured	
Hyalohyphomycosis	16 (54.8)
*Aspergillus* spp.	7 (22.6)
*Fusarium* spp.	5 (16.1)
*Curvularia* spp.	2 (6.5)
*Scedosporium* spp.	1 (3.2)
*Penicillium* spp.	1 (3.2)
Zygomycosis	7 (22.6)
*Rhizopus* spp.	3 (9.7)
*Mucor* spp.	2 (6.5)
*Lichtheimia* spp.	1 (3.2)
*Cunninghamella* spp.	1 (3.2)
Other	2 (6.5)
*Alternaria* spp.	1 (3.2)
*Rigidoporus* spp.	1 (3.2)
Morphology identified on histopathology only	6 (19.4)
Suggestive of zygomycosis	3 (9.7)
Suggestive of hyaline mold	2 (6.5)
Antifungal prophylaxis at time of IFS diagnosis	
None	29 (93.6)
Voriconazole	2 (6.5)
Antifungal treatment during course of infection^b^	
Amphotericin B (liposomal)	30 (96.8)
Voriconazole	22 (71.0)
Isavuconazole	12 (38.7)
Posaconazole	11 (35.5)
Terbinafine	8 (25.8)
Micafungin	8 (25.8)
Caspofungin	4 (12.9)
Flucytosine	1 (3.2)
Olorofim	1 (3.3)
Fosmanogepix	1 (3.3%)

Abbreviation: IFS, invasive fungal disease.

^a^Patients could be on >1 antifungal treatment.

^b^Percentages are reported for the n = 28 subset in whom galactomannan values were assessed.

Half of cases were unilateral (51.6%), with further anatomical and surgical characteristics described in Table 4. The most prevalent characteristics were necrotic tissue (45.2%) on sinonasal endoscopy and mucosal thickening (87.1%) on CT scan. Orbital extension (25.8%) and intracranial extension (9.7%) were seen in a small fraction of cases, with all patients with intracranial extension also experiencing orbital extension. In this subset of cases, mortality by 6 months was 71.4% for the orbital extension group (n = 7 with available death dates) and 50% for the intracranial group (n = 2 with available death dates). Serum fungal biomarkers were infrequently positive, with (1,3)-ß-D-glucan detected in 19.4% of patients and galactomannan in 9.7%. Most patients underwent functional endoscopic sinus surgery (FESS; 80.0%), while a smaller fraction of the cohort underwent multiple FESS interventions (26.7%). The maxillary sinuses (76.0%) were the most common anatomic site for surgical intervention, and middle turbinate resection (52.0%) was the most commonly performed nasal procedure. The median time from IFS diagnosis to surgery (IQR) was 1 (0–2) day. Stratified analyses characterized differences in 6-month mortality between subgroups. Patients receiving novel antifungals (n = 13) had a 46.2% 6-month mortality, as compared with 38.9% in those not receiving novel antifungals (n = 18; *P* = .69). There were minimal differences in most demographic, endoscopic/imaging, and mortality outcome characteristics between patients who received newer vs older antifungal drugs (Supplementary Table 1). Acute myeloid leukemia was more prevalent in the newer antifungal group (92.3%) as compared with the older antifungal group (50.0%; *P* = .02).

**Table 4.** Radiographic, Sinonasal Endoscopic, and Surgical Data

Characteristic	Value^a^
Overall, No.	31
CT scan closest to time of IFS diagnosis	
Laterality, No. (%)	
Unilateral	16 (51.6)
Bilateral	11 (35.5)
Radiographic features, No. (%)	
Mucosal thickening	27 (87.1)
Sinus involvement	26 (83.9)
Nasal cavity opacification	17 (54.8)
Fat stranding	8 (25.8)
Orbital extension	8 (25.8)
Bony erosion	6 (19.4)
Intracranial extension	3 (9.7)
Lund-McKay score, median (IQR)	9.0 (4.0–12.5)
Sinonasal endoscopy and physical exam findings, No. (%)	
Necrotic tissue	14 (45.2)
Mucosal edema	8 (25.8)
Crusting	8 (25.8)
Cranial nerve involvement^b^	6 (19.4)
Pale mucosa	4 (12.9)
Purulence	2 (6.5)
No significant findings	3 (9.7)
Ulceration	1 (3.2)
Surgical intervention via functional endoscopic sinus surgery, No. (%)^c^	25 (80.6)
Unilateral sinuses	18 (72.0)
Bilateral sinuses	6 (24.0)
Septum only	1 (4.0)
Anatomical region of operation, No. (%)	
Paranasal sinuses	
Maxillary	19 (76.0)
Anterior ethmoids	16 (64.0)
Posterior ethmoids	15 (60.0)
Sphenoid	6 (24.0)
Frontal	5 (20.0)
Medial maxillectomy	2 (8.0)
Nasal structures	
Middle turbinate resection	13 (52.0)
Inferior turbinate resection	8 (32.0)
Septoplasty	6 (24.0)
Septal resection	5 (20.0)
Lateral nasal wall	2 (8.0)
Other	
Lamina papyracea	2 (8.0)
Orbit	1 (4.0)
Multiple FESS operations, No. (%)	7 (22.6)
Timing of surgical intervention^d^	
Median time from IFS diagnosis to surgical intervention, median (IQR), d	1 (0–2)
≤1 d of IFS diagnosis, No. (%)	15 (65.2)
>1 d of IFS diagnosis, No. (%)	8 (25.8)

Abbreviations: CT, computed tomography; FESS, functional endoscopic sinus surgery; IFS, invasive fungal disease; IQR, interquartile range.

^a^For percentages that do not tally to 100%, unknown variable values comprise the deficit.

^b^All n = 6 patients with cranial nerve involvement had CNV (trigeminal) involvement.

^c^Percentages for surgical data are reported as a percentage of the subset of n = 25 patients who received surgical intervention.

^d^Percentages for timing data are reported as a percentage of the subset of n = 23 patients who had recorded dates.

Patients with *Aspergillus* IFS (n = 7) had a 14.3% 6-month mortality, as compared with 50.0% in the non-*Aspergillus* IFS group (n = 24; *P* = .19). Patients undergoing early surgical intervention within 1 day of IFS diagnosis (n = 15) had a 26.7% 6-month mortality, as compared with 50.0% in the delayed surgical intervention after 1-day group (n = 8; *P* = .37). Patients undergoing a hematopoietic stem cell transplant (n = 16) had a 50.0% 6-month mortality, as compared with 33.3% in those without a HCT transplant (n = 15; *P* = .35). Patients receiving systemic steroids (n = 7) had a 14.3% 6-month mortality, as compared with 52.2% of those not receiving systemic steroids (n = 23; *P* = .19).

## DISCUSSION

In our descriptive case series of 31 proven IFS cases in patients with hematologic malignancies—most of whom had AML, had marked reductions in all cell lines, were treated initially with liposomal amphotericin B but did not receive antifungal prophylaxis before diagnosis, and underwent surgical intervention—we identified a high overall mortality commensurate with the previous literature and no differential effect of newer-generation antifungal agents.

The majority of hematologic malignancies represented in our cohort were AML, consistent with numerous previous reports [28, 29]. The data show that most patients were not on mold prophylaxis, consistent with our institution's practice (no antifungal prophylaxis for patients with AML or allogeneic hematopoietic stem cell transplantation). Our finding that *Aspergillus* and *Mucorales* species were the most common molds identified has been echoed in previous studies [28–30]. We identified rare molds in our cohort, including *Curvularia*, *Scedosporium*, *Penicillium*, *Lichtheimia*, *Cunninghamella*, *Alternaria*, and *Ridigoporus* species, which have been similarly uncommon in other cohorts and primarily found in isolated case reports [3, 31, 32].

Subgroup analyses were all univariable (no multivariable adjustment) and were substantially limited by issues such as small sample size in subgroups and sensitivity to single events. Nonetheless, these analyses are presented for descriptive and hypothesis-generating purposes in an undercharacterized population [33]. Despite insignificant Fisher exact tests, our analysis found the largest qualitative 6-month survival differences for the non-*Aspergillus* group (compared with *Aspergillus* IFS) and for the nonsteroids group (compared with those receiving steroids).

Use of newer antifungal agents in aggregate (including 12 cases treated with isavuconazole and 1 with olorofim) was not associated with improved prognosis, despite in vitro and in vivo evidence suggesting a benefit for next-generation agents like olorofim [20, 34, 35] and fosmanogepix [36–38]. We note that our definition of newer antifungals grouped together isavuconazole (an azole) with olorofim, which have different mechanisms of action, due to small sample size. Another limitation is that our analytic groups are not strictly mutually exclusive, as all patients on newer agents received first-line amphotericin B per institutional protocols before being switched to newer therapy. Small sample size hampers our findings, but could also be explained by potential selection bias, where those with more advanced malignancy, comorbidities, or resistant fungal isolates received newer agents. It is also possible that new classes of antifungal drugs may not be able to overcome advanced IFS in the presence of heavily immunocompromised states. However, although overall mortality was high, the single patient treated with fosmanogepix (for disseminated fusariosis) did exhibit prolonged survival. Studies with a larger number of patients on newer-generation therapies are needed.

Necrosis on sinonasal endoscopic exam was a prominent finding in our cohort, similar to other descriptions [29]. Because necrosis commonly localizes to the middle turbinate, our results support the utility of early middle turbinate biopsy for high-suspicion cases while avoiding unnecessary risk of hemorrhage in patients with thrombocytopenia [9]. Although patients with orbital/intracranial involvement of IFS comprised a small subset in our study, they had a poor 6-month survival of 71.4% for orbital extension and 50.0% for intracranial extension. This accords with much of the available literature that has identified orbital and intracranial extension as being associated with poor survival within 6 months [7, 39, 40]. Potential differences may be due to small sample size, referral bias, and earlier diagnosis of cases before extensive orbital extension in our institution as compared with historical cohorts.

Serum fungal biomarkers are often used in clinical practice to assist with diagnosis of fungal infections. The median absolute neutrophil count (ANC) in our cohort was 0.01, supporting current evidence that ANC be used as a diagnostic aid for IFS workup and monitoring [18, 41] and consistent with previous reports of prolonged neutropenia associated with higher IFS mortality rates [28]. However, less than a quarter of serum (1,3)-ß-D-glucan assays (19.4%) sent at IFS diagnosis in our cohort yielded a positive result, despite high reported sensitivity [42] and a significant fraction of molds being known to contain (1,3)-ß-D-glucan in their cell walls. Similarly, only a small fraction of patients had a positive galactomannan index (9.7%), despite decent reported sensitivity as a diagnostic tool for IFS in past studies [43, 44]. Our results may be explained by a relatively high prevalence of proven or suggestive zygomycosis infections (32.3%), where cell walls do not contain these 2 polysaccharides, or by a high prevalence of localized infection (54.8%) rather than disseminated infection, in which cell wall components do not seep into the bloodstream. This suggests that we detected IFS cases early before vascular involvement. This underscores the low sensitivity and limited diagnostic utility of these biomarkers for IFS; IFS diagnosis should therefore not be excluded on the basis of seronegative markers alone. Although histopathologic evaluation of biopsy tissue confirmed IFS diagnosis in the majority of cases, multiple biopsies were often needed for diagnosis. Importantly, antifungal susceptibility testing can only be completed on specimens identified by culture. These findings suggest room for diagnostic improvement in patients with IFS, particularly the need for rapid next-generation molecular diagnostics (eg, metagenomic sequencing, PCR) and new high-fidelity biomarkers.

Clinical diagnosis of IFS involves a physician's clinical suspicion in the context of a high-risk population. Earlier diagnosis of IFS leads to faster surgical and medical antifungal interventions [45]. Delayed surgical intervention [46] and delayed initiation of systemic antifungal therapy [47] are both key negative prognostic indicators for patients with IFS. Early surgical intervention improves IFS mortality outcomes [46, 48], a finding that our study preliminarily and qualitatively supports through our exploratory subgroup analysis (despite a nonsignificant Fisher exact test due to small sample size), given that extensive and continued debridement debulks infectious burden and increases penetrative ability of antifungal drugs to slow disease progression [49]. The vast majority of patients in our cohort underwent early surgical intervention, occurring at a median of 1 day from IFS diagnosis, in contrast to other studies where the median interval between diagnosis and surgical debridement was 10+ days [24, 50]. However, the baseline characteristics of the cohorts likely differed, with our cohort having more advanced malignancy with resulting neutropenia. The mortality rates seen in our cohort from 1 month to 1 year after surgical debridement were similar to previous studies of IFS in patients with hematological malignancies [28, 29].

Otolaryngology subspecialty consultation is a key component of IFS management, and the involvement of a fellowship-trained rhinologist has been shown to improve survival given their unique skill set of endoscopic surgery and sinonasal endoscopic examination [3]. Indeed, the surgical expertise and the ability to differentiate diseased from healthy mucosa enable full resections of necrotic tissue in IFS and can lead to improved prognosis [51].

### Strengths and Limitations

Our use of proven/definite IFS captured cases with a higher degree of certainty as compared with previous studies using “probable” IFS. An integrated, interdisciplinary approach is necessary to manage patients with hematological malignancies with IFS. Thus, prognosis and risk of IFS progression and outcomes must assess host characteristics as well as the antifungal and surgical interventions in combination. To our knowledge, few previous reports in the IFS literature have captured the oncologic treatment, diagnostics, antifungal treatment, and anatomical characteristics of proven IFS to this degree of detail. Our work also characterizes newer-generation antifungal therapies and novel oncologic therapies in IFS patients that have previously been underexplored. Our study integrates a descriptive design with cross-group comparisons. Other strengths include the number and depth of chart-reviewed variables, extensive collection of radiographic data, and multireader verification.

There are limitations to the current study. Our smaller sample size (n = 31) limits statistical power and the inferences able to be made, which can be particularly restrictive when outcomes are compared across stratified groups. We were unable to control for potential confounders (eg, calendar time effects, secular trends, changes in treatment strategies or protocols) in both the main analyses and subgroup analyses due to sample size. Despite data being collected from a tertiary academic institution capturing a large geographic catchment area, our inferences may potentially be less generalizable than multi-institutional or pooled studies. Future studies with a larger sample size should capture the impact of newer-generation antifungals on IFS survival.

## CONCLUSIONS

Despite novel antifungal therapies and aggressive early interventions, patients with hematological malignancies and IFS had high mortality. Our findings emphasize the critical illness and immunosuppressed state of patients at the time of IFS diagnosis and how progressive malignancy or relapsed disease, as well as other host immune factors, is intrinsically linked with patients’ lack of ability to control fungal disease. Future studies should compare IFS cases in the hematologic malignancy population with non-IFS controls to assess specific risk factors of prognosis and mortality. Identification of specific prognostic factors that portend positive outcomes for patients with IFS may help lead to improved strategies for prevention and/or treatment of this severe disease.

## Supplementary Material

ofag304_Supplementary_Data

## Supplementary Data

Supplementary materials are available at *Open Forum Infectious Diseases* online. Consisting of data provided by the authors to benefit the reader, the posted materials are not copyedited and are the sole responsibility of the authors, so questions or comments should be addressed to the corresponding author.
