# Supplementary material for: Invasive Fungal Sinusitis in Patients With Hematological Malignancies: A 20-Year Study From a Tertiary Academic US Hospital System
Source: Open Forum Infect Dis. 2026 May 15;13(6):ofag304. doi: 10.1093/ofid/ofag304 (PMC13229113; doi:10.1093/ofid/ofag304)

**Supplemental Material**

*Table S1.* Select differences between newer vs. older antifungal groups

| **Characteristic** | **Newer Antifungal Group ^1^** | **Older Antifungal Group ^2^** | **P-value ^3^** |
| --- | --- | --- | --- |
| Overall *N* | 13 | 18 |  |
| Demographics |  |  |  |
| Age (years), *median* (IQR) | 58 (50–68) | 60 (54–64) | 0.61 |
| Male, *n* (%) | 6 (46.2) | 10 (55.6) | 0.72 |
| BMI (kg/m^2^), *median*  (IQR) | 23.7 (22.6–28.4) | 22.9 (19.2–28.9) | 0.70 |
| Oncologic, *n* (%) |  |  |  |
| Acute myeloid leukemia | 12 (92.3) | 9 (50.0) | 0.02 |
| Myelodysplastic syndrome | 1 (7.7) | 6 (33.3) | 0.19 |
| Receipt of newer  generation anti-cancer  therapy ^4^ | 8 (61.5) | 8 (44.4) | 0.47 |
| Endoscopy or imaging, *n* (%) |  |  |  |
| Bilateral involvement | 4 (30.8) | 7 (38.9) | 0.69 |
| Necrotic tissue | 6 (46.2) | 8 (44.4) | 1.00 |
| Bony erosion | 2 (15.4) | 4 (22.2) | 0.56 |
| Orbital extension | 4 (30.8) | 4 (22.2) | 0.69 |
| Intracranial extension | 2 (15.4) | 1 (5.6) | 0.75 |
| Disseminated extent of  infection | 4 (30.8) | 10 (55.6) | 0.27 |
| Outcomes |  |  |  |
| Overall mortality, *n* (%) | 10 (76.9) | 12 (66.7) | 0.42 |
| Time from diagnosis to  death (days), *median* (IQR) | 134 (75–307) | 31 (18–226) | 0.34 |
| Time from diagnosis to  surgery (days), *median*  (IQR) | 2 (1–2) | 1 (0 – 2) | 0.50 |

^1^ Newer antifungal group is defined as those individuals receiving medications approved by the FDA in 2015 or later or still in unapproved investigational status, including olorofim, fosmanogepix, or isavuconazole. Categorical variables are depicted as n (%), while continuous variables are displayed as median (IQR).

^2^ Older antifungal group is defined as those receiving medications approved by the FDA prior to 2015, including amphotericin B, micafungin, voriconazole, posaconazole, terbinafine, or other. Categorical variables are depicted as n (%), while continuous variables are displayed as median (IQR).

^3^ Fisher’s exact test was used to compare categorical variables where at least one cell had a value less than 5.
T-tests or Wilcoxon rank-sum tests (Mann-Whitney U tests) were used to compare continuous variables.

^4^ Defined as targeted therapy, tyrosine kinase inhibitor, or antibody-based immunotherapy.

*Figure S1.* Distribution of single-institution IFS cases by year of diagnosis


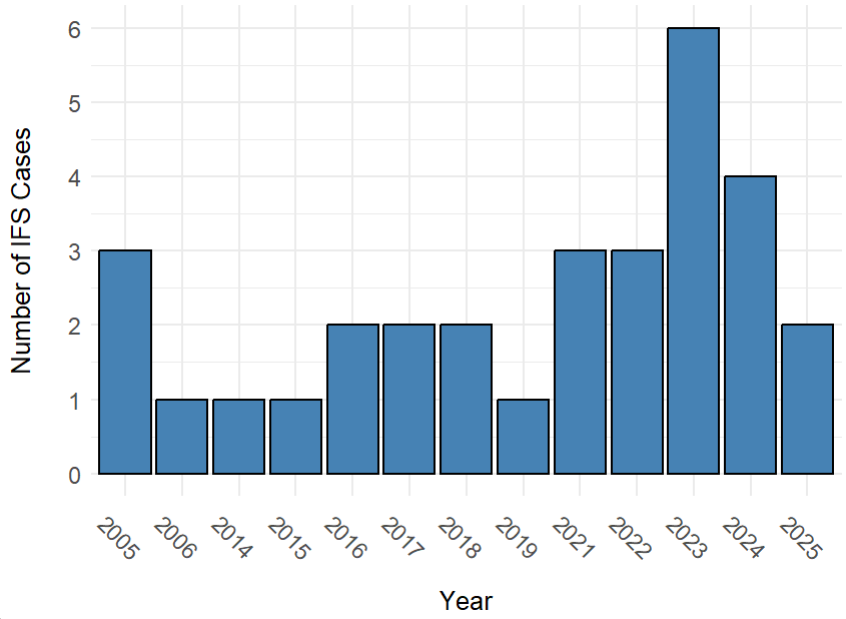


*Figure S2.* Estimated Kaplan-Meier survival curve for time since IFS diagnosis to death in months


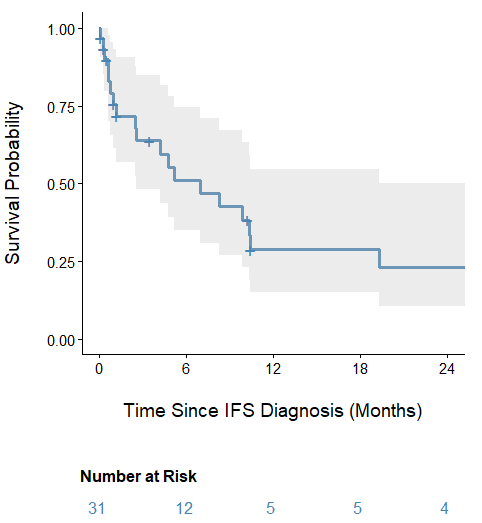

Supplement: ofag304_Supplementary_Data [file ofag304_supplementary_data.docx]
